# Factors associated with smoking among adolescent males prior to incarceration and after release from jail: a longitudinal study

**DOI:** 10.1186/1747-597X-8-37

**Published:** 2013-10-31

**Authors:** Megha Ramaswamy, Babalola Faseru, Karen L Cropsey, Marvia Jones, Karisa Deculus, Nicholas Freudenberg

**Affiliations:** 1 Department of Preventive Medicine and Public Health, University of Kansas School of Medicine, 3901 Rainbow Boulevard, MS 1008, Kansas City, Kansas 66160, USA; 2 Department of Psychiatry, University of Alabama at Birmingham School of Medicine, 1702 2nd Avenue. S. FOT 1203, Birmingham, Alabama 35294, USA; 3 Department of Applied Behavioral Science, University of Kansas, 4001 Dole Human Development Center, 1000 Sunnyside Avenue, Lawrence, Kansas 66045, USA; 4 Children’s Mercy Hospitals and Clinics, 2401 Gillham Road, Kansas City, Missouri 64108, USA; 5 School of Public Health at Hunter College, City University of New York, 2180 3rd Avenue, New York City, New York 10035, USA

**Keywords:** Adolescents, Smoking, Incarcerated smokers, Re-entry

## Abstract

**Background:**

The prevalence of cigarette smoking among incarcerated adult men and women is three-four times higher than in the general population, ranging from 70-80%. However, little is known about factors associated with smoking among incarcerated adolescents, especially upon their re-entry into communities after release from jail. The current study explores factors associated with smoking among adolescent males prior to incarceration and one year after their release from jail.

**Methods:**

We conducted a secondary data analysis of the Returning Educated African-American and Latino Men to Enriched Neighborhoods (REAL MEN) study, which was designed to reduce HIV risk, substance use, and recidivism among 16–18 year old males leaving jail. We examined differences between smokers and non-smokers at the time of their incarceration (N = 552) and one year after their release from jail (N = 397) using t-tests and chi-square tests. Using logistic and linear regression we examined factors associated with current smoking status, frequency of smoking, and quantity of cigarettes smoked per day both prior to the young men’s incarceration and one year after their release from jail.

**Results:**

Prior to incarceration, 62% of the young men reported smoking, and one-year after jail release, 69% reported smoking. Prior to incarceration, foster care history, not living with parents, not attending school, drug sales, number of sex partners, gang involvement, current drug charges, and number of prior arrests were positively associated with smoking indicators prior to incarceration. Having violent charges was inversely associated with smoking indicators prior to incarceration. One-year after release from jail, foster care history and number of prior arrests before the index incarceration were associated with smoking indicators.

**Conclusions:**

Several problem behaviors may be associated with adolescent males’ smoking behaviors prior to incarceration. However, the young men’s histories of difficult life circumstances and engagement in illegal activity may have long-term consequences on smoking for these young men during their transition between jail and community. Findings suggest a need for comprehensive risk reduction interventions in settings in which disadvantaged young men are institutionalized, starting in childhood.

## Background

The prevalence of tobacco cigarette smoking among incarcerated men and women is three to four times higher than in the general population, ranging from a rate of 70% to 80% for both adult male and female inmates [1,2]. While the rate of current smoking among adolescents in the general population is 23% [3], the literature suggests that smoking rates among incarcerated adolescents are substantially higher. Cropsey and colleagues [4] found that about half of young people in juvenile justice facilities reported smoking every day prior to incarceration, whereas only about 9% of adolescents in the general population report being frequent smokers [3]. To date, few researchers have examined the factors associated with smoking in this high-risk group of incarcerated adolescents.

In the general population, problem behavior theory suggests that adolescent risk behaviors, such as drug use, sex risk, and delinquency, for example, cluster [5], and may explain smoking behaviors [6-9]. Indeed some of these same factors, like illicit drug use, explain smoking behaviors in at least one study of adolescents in juvenile detention centers [4]. In later iterations of problem behavior theory [10], authors argue that the expression of adolescent risk behaviors rests in the balance of both risk and protective barriers. These protective factors, such as living with parents and reward for prosocial behavior in school, have also been associated with smoking behaviors among adolescents [4,6].

Thus, for the present study, we examined behavioral risks that may be associated with smoking, for example, risk for alcohol and drug use, sex risk, violence, and delinquency. We also focused on the absence of protective factors in incarcerated adolescents’ social environment, that is, their housing stability, ties to family, peers, school, employment, and health care. Problem behavior theory would have us study the role of psychosocial variables as protective factors [10], e.g. support of parents and peers for pro-social behavior. In part because this was a secondary data analysis, we used measurements available to us – those of structural protective factors. These stand in as proxies for psychosocial variables that may act as “controls” for problem behaviors. Given the difficult life circumstances of many youth involved in the criminal justice system – for example only about three-quarters of incarcerated youth live with their parents [4,11] and half experience reincarceration within one year [12]-we thought examining behavioral risks and lack of protective factors would help explain our sample’s smoking behaviors and also provide new directions for interventions aimed at preventing smoking. Such an analysis is also justified given the importance of these variables to health and social outcomes after release from jail [13-16].

Our study is one of the first that tracks 16–18 year olds as they enter jails, and then follows them upon their release into the community, allowing us to understand how behaviors and life circumstances change over time. We hypothesize, specifically, that though a cluster of problem behaviors in adolescence may explain smoking behaviors prior to incarceration, upon reentry into their communities after jail, factors such as housing stability, ties to family, peers, school, employment, healthcare, and the experience of re-incarceration may also be important factors that are associated with smoking for young adults with the unique risk of a criminal justice history [13-16].

This is also one of the a few studies that focuses on the lives and smoking behaviors of a sample that is over 90% Black and Latino. Youth of color are disproportionately represented in the justice system in New York City [17,18], a jurisdiction that incarcerates 16–18 year olds in adult institutions, not juvenile detention centers. Few studies have investigated the effect of interventions on smoking rates among minority adolescents, even in the general population. For example, a recent study by Branstetter and colleagues [19] recruited a non-adjudicated youth sample that was 75% White. Other intervention studies in the general population are similarly administered with mostly White participants [20,21], making generalization difficult, both based on race/ethnicity and adjudication status. The results of this study will hopefully shed light on areas for intervention with a unique sample of mostly minority youth who are incarcerated in adult jails.

The objectives of this paper were two-fold; first, we described the characteristics of young men prior to their incarceration and one year after their release from jail, stratified by their smoking status. Secondly, using theory of problem behavior and an understanding of our sample’s difficult life circumstances as a guide for variable selection, we assessed factors associated with smoking behaviors among our sample of young men as they entered jail and upon their reentry into the community. The goal of our study was to inform the development of smoking cessation interventions for this group of men as they transition from an adolescence spent in jail to navigating their communities into adulthood.

## Methods

### Participants

This paper was a secondary data analysis of the *Returning Educated African*-*American and Latino Men to Enriched Neighborhoods* (REAL MEN) study, which was designed to reduce HIV risk, substance use, and recidivism for incarcerated young men in New York City. We recruited participants from two facilities, located at the New York City Department of Correction’s Rikers Island Detention Center, that house all male adolescent inmates in New York City. All interviews were conducted from 2002–2007 and eligible males16-18 years old were recruited in the study, which was approved by the Hunter College, City University of New York, and New York City Department of Health and Mental Hygiene Institutional Review Boards. The study and evaluation of the intervention have been described in detail elsewhere [11,17].

### Interviews

Five hundred fifty-two young men completed intake interviews, with interview questions referring to circumstances and behaviors prior to incarceration. These Time 1 interviews were conducted in the jail by project staff, and at the completion of the interview, participants were randomly assigned to receive a single jail-based discharge planning session or a 30-hour intervention that began in jail and continued into the community after release, with a total of eight sessions. The intervention was informed by an intersectional approach to HIV prevention, taking into account the intersecting oppressions of race, class, and gender and the young men’s criminal justice status [11,17]. The intervention did not directly address tobacco use. The REAL MEN project contracted with The Center for Urban Epidemiologic Studies (CUES) at the New York Academy of Medicine to conduct follow-up Time 2 interviews with enrolled participants at approximately 12 months after release from jail. The Time 2 interview was completed by 397 participants. The majority of participants completed the follow-up interview in a CUES office, while others completed the interview in a New York City jail or state prison, by telephone, or at some other location. To conduct an attrition analysis, we examined 27 variables in the following categories: sociodemographics, living situation, employment, education, mental health/physical health status, alcohol/drug risk, sex risk, and criminal justice background. Participants who completed the follow-up interview were more likely to have reported at baseline that they lived with parents or a legal guardian, had fewer status violation charges, and more diagnoses of asthma (p ≤ 0.05). There were no differences based on smoking status at baseline between participants who completed the follow-up interview and those who did not.

### Variables

The dependent variables of interest were three measures of smoking behavior: 1) We assessed whether or not each participant was a current smoker at the Time 1 interview and again at the Time 2 follow-up interview with this question: “Do you smoke tobacco cigarettes?” 2) To assess frequency of smoking, at Time 1 we asked participants, “In the 30 days prior to this incarceration, how many *days* did you smoke tobacco cigarettes?” At Time 2, we asked about smoking in the past 90 days. Ninety days was used as a measure at the Time 2 interview because the parent study was concerned with post-release behavior for a three month period. At Time 1 we were interested in behaviors immediately prior to incarceration, so most questions referred to the period of 30 days prior to incarceration. 3) We assessed quantity of cigarettes smoked by asking the question “In the 30 days prior to this incarceration (past 90 days at Time 2), approximately how many *cigarettes* did you smoke a day?”

We examined independent variables in the following domains at the Time 1 and Time 2 interviews: demographics, housing stability, social ties, health insurance, employment, education, alcohol and drug use, sex risk, interpersonal violence risk, criminal justice background, and receipt of the REAL MEN intervention. We chose independent behavioral risk variables based on problem behavior theory and studies of smoking among adolescents [6-9]. We also added additional variables that measured housing stability, ties to family, peers, school, employment, health care, and the experience of re-incarceration based on studies that have documented the importance of these indicators of stability to health, wellness, and social outcomes after release from jail [13-17]. Independent variables are described in Table 1.

**Table 1:** Independent variable descriptions

	**Assessed at time 1**	**Assessed at time 2**
	**Refers to period *prior to arrest***	**Refers to *current situation***
Demographics		
Age	Date of birth	Date of birth at Time 1 plus number of days elapsed between Time 1 and 2 interviews
Race/ethnicity	White, Black, Asian/Pacific Islander, American Indian or Alaska native, Bi-racial, Other; as Hispanic. Categories collapsed to make Hispanic ethnicity a mutually exclusive category if overlap with other race identity	--
Housing stability		
Foster care history	Ever having been in New York City’s Administration for Children’s Services, a group home, or foster care	--
Living with parents or legal guardian	Living with parents, legal guardian, or other relatives.	Living with parents, legal guardian, or other relatives
Unstably housed	Living in a shelter, from place-to-place, homeless, on the streets, in an empty building, or in an institution	Living in a shelter, from place-to-place, homeless, on the streets, in an empty building, or in an institution
Social ties	Number of people in their lives felt close to	--
No health insurance	Not paying for medical care in the past year with Medicaid or other health insurance	Not paying for medical care in the past year with Medicaid or other health insurance
Unemployed	Being unemployed and looking/not looking for work	Being unemployed and looking/not looking for work
Education		
Ever held back in school	Ever having stayed a grade back in school	--
Not attending school regularly	Being enrolled in school but not attending most of the time, suspended or expelled from school, dropped out of school, or graduated from school	Being enrolled in school but not attending most of the time, suspended or expelled from school, dropped out of school, or graduated from school
Lifetime learning disability diagnosis	Ever having been told by a guidance counselor, social worker, physician, or psychologist that they had a learning disability, Attention Deficit Disorder (ADD), or hyperactivity	--
Alcohol and drug risk		
Alcohol use	Number of times a week on average alcohol consumed in past 30 days	Number of times a week on average alcohol consumed in past 90 days
Marijuana use	Number of days marijuana used in past 30 days	Number of days marijuana used in past 90 days
Hard drug use	Number of days cocaine, crack, heroin, inhalants, acid, ecstasy, downers, speed, PCP, or steroids used in past 30 days	Number of days cocaine, crack, heroin, inhalants, acid, ecstasy, downers, speed, PCP, or steroids used in past 90 days
Sold drugs to get money for drugs/alcohol	Selling drugs to get money to pay for purchase of drugs/alcohol for personal use in past 30 days	Selling drugs to get money to pay for purchase of drugs/alcohol for personal use in past 90 days
Drug/alcohol dependence	Participants asked 6 questions about drug/alcohol use in the past year, such as: “Did you need to use more drugs or alcohol to get the same high as when you first started using?” If participants answered “yes” to 6 out of 6, classified as “drug/alcohol-dependent” according to DSV IV criteria [22]	Participants asked 6 questions about drug/alcohol use in the past year, such as: “Did you need to use more drugs or alcohol to get the same high as when you first started using?” If participants answered “yes” to 6 out of 6, classified as “drug/alcohol-dependent” according to DSV IV criteria [22]
Sex risk		
Sex partners	Number of sex partners in past three months	Number of sex partners in past three months
Inconsistent condom use	Not “always” using condoms with all sex partners in past three months	Not “always” using condoms with all sex partners in past three months
Interpersonal violence risk		
Gang involvement	Ever having been involved in a gang, for example with the “Bloods”, “Crips”, or “Latin Kings”	--
Weapons possession during illegal activity	Carrying a gun, knife, or any other type of weapon while engaging in illegal activities in last year	Carrying a gun, knife, or any other type of weapon while engaging in illegal activities in last year
Criminal justice background		--
Incarcerated for drug charges	Having a current charge for sale, manufacturing, use, or possession of drugs/controlled substances	--
Incarcerated for violent charges	Having a current charge for armed robbery, possession of a weapon or weapons charge, offenses against family, children, reckless endangerment of children, domestic violence, sex offenses other than rape or prostitution, or simple assault	--
Prior arrests	Number of times ever arrested	--
Arrests after release from jail	--	Number of arrests since release from jail/enrollment in REAL MEN
Went back to jail after index incarceration	--	Whether past year’s arrests led to an incarceration

Though REAL MEN was an HIV prevention intervention designed to reduce HIV risk, illicit substance use, and recidivism among young men leaving jail, it did not specifically address tobacco use. However, in the multivariate models to assess smoking behavior at Time 2 (post-intervention for those randomized to the intervention) we created a control variable that measured intervention assignment at baseline.

### Data analysis

The research team stratified participants’ characteristics by Time 1 and Time 2 smoking status. We identified significant differences between self-reported smokers and non-smokers by using *t*-tests for mean differences for continuous variables or Mann–Whitney U tests where variables were not normally distributed. Chi-square tests of independence were used for dichotomous variables or Fisher’s Exact tests for variables with cells where N < 5. Dependent variables were smoking status (whether participants self-reported being smokers or non-smokers), frequency of smoking (number of days smoked), and quantity of cigarettes smoked (number of cigarettes smoked per day). Independent variables were social and behavioral characteristics of the sample. Logistic regression was used to test associations between social and behavioral characteristics of the young men with smoking status. Variables for the logistic regression models were based on bivariate associations with smoking status at the p ≤ 0.01 level. Linear regression was used to test associations between characteristics of the young men with frequency of smoking and quantity of cigarettes smoked. Similarly, variables for the linear regression models were based on bivariate associations with smoking status at the p ≤ 0.01 level. All analyses were conducted with SPSS Version 20 for Mac (SPSS Inc., Chicago, Ill).

## Results

### Participants

All participants in the REAL MEN study were male, and the mean age at the Time 1 interview was 17.99 years (SD: 0.71 years). One year after release from jail, the mean age of participants was 19.60 years (SD: 0.93 years), suggesting that on average, young men spent seven months in jail prior to release. The majority of the sample was Black (55.8%) and Latino (38.1%). Participant characteristics by smoking status prior to incarceration and one year after release from jail are described in Table 2.

**Table 2:** Characteristics of young men in jail and one year after release, by smoking status

	**Prior to incarceration, N = 552**		**One year after release from jail, N = 397**	
	**Smokers**	**Non-smokers**	**Smokers**	**Non-smokers**
	**N (%)**	**N (%)**	**N (%)**	**N (%)**
	**344 (62.3)**	**208 (37.7)**	**274 (69.2)**	**122 (30.8)**
Age, mean (sd)	18.04 (0.70)	17.89 (0.75)	19.68 (0.93)	19.43 (0.93)
(Time 1 d.f. = 550; Time 2 d.f. = 391)				
Black†	184 (53.6)	132 (63.5)	149 (54.4)	72 (59.5)
Latino†	140 (40.8)	66 (31.7)	109 (39.8)	41 (33.9)
Foster care history†	113 (33.0)*	34 (16.3)	87 (31.9)*	19 (15.6)
Not living with parents or legal guardian	105 (30.5)*	35 (16.8)	101 (37.4)	47 (38.8)
Unstably housed	11 (3.2)	3 (1.4)	72 (26.7)	25 (20.7)
People felt close to in past year, mean (sd)	4.96 (7.67)	4.99 (5.05)	5.59 (6.35)	5.49 (7.17)
(Time 1 d.f. = 498; Time 2 d.f. = 394)				
No health insurance	32 (9.6)	14 (7.1)	67 (26.8)	20 (18.0)
Unemployed	218 (63.4)	135 (64.9)	292 (73.5)	78 (65.5)
Ever held back in school†	167 (48.7)	90 (43.3)	143 (52.2)	48 (39.3)
Not attending school regularly	242 (71.6)*	124 (60.5)	218 (80.1)	96 (78.7)
Lifetime learning disability diagnosis†	80 (23.3)	40 (19.4)	64 (23.4)	25 (20.5)
Days in past 30/90‡ smoked cigs., mean (sd)	24.99 (9.68)	0.00 (0.00)	70.79 (31.80)	0.00 (0.00)
Cigarettes smoked each day, mean (sd)	10.66 (10.75)	0.00 (0.00)	13.41 (35.47)	0.00 (0.00)
Times used alc. in wk. in 30/90‡, mean (sd)	2.72 (4.19)	2.31 (6.47)	3.97 (9.31)	5.12 (13.04)
(Time 1 d.f. = 356; Time 2 d.f. = 263)				
Days in past 30/90‡ used marij., mean (sd)	23.22 (11.50)	20.30 (12.16)	50.37 (39.93)	58.13 (37.41)
(Time 1 d.f. = 419; Time 2 d.f. = 110)				
Hard drug use in past 30/90‡ days	28 (8.2)	8 (3.9)	32 (11.7)	13 (10.7)
Sold drugs to get money for drugs/alcohol	181 (58.2)*	68 (45.3)	41 (15.0)	13 (10.7)
Drug/alcohol dependence in past year	85 (24.7)	38 (18.3)	54 (22.5)	10 (11.5)
Sex partners in past 3 mo., mean (sd)	4.42 (7.88)*	3.01 (3.13)	0.63 (3.37)	0.24 (0.94)
(Time 1 d.f. = 484; Time 2 d.f. = 351)				
Inconsistent condom use in past 3 mo.	240 (71.6)	119 (63.0)	128 (76.6)	60 (82.2)
Gang involvement†	95 (27.6)*	38 (18.3)	76 (27.7)	22 (18.0)
Weapons possession during illegal activity	222 (69.2)	131 (66.5)	80 (29.4)	32 (26.7)
Incarcerated for drug charges†	119 (34.8)*	43 (20.7)	79 (28.9)	30 (24.6)
Incarcerated for violent charges†	100 (29.2)*	104 (50.0)	93 (34.1)	59 (48.4)
Prior arrests, mean (sd)†	5.79 (6.24)*	4.29 (4.97)	5.79 (6.38)*	4.29 (4.39)
(Time 1 d.f. = 546; Time 2 d.f. = 328)				
Arrests after release from jail, mean (sd)	--	--	1.51 (1.59)	1.24 (1.57)
(Time 2 d.f. = 389)				
Went back to jail after index incarceration	--	--	138 (51.1)*	46 (37.7)
Received REAL MEN intervention	--	--	138 (50.4)	58 (47.5)

*p ≤ 0.01 for comparison of smokers vs. non-smokers, Pearson’s Chi-Square Test (d.f. = 1). For variable “unstably housed” prior to incarceration, Fisher’s Exact Test, p = 0.75. For continuous variables where means and standard deviations were reported, Independent Samples T-Tests were performed. Degrees of freedom are notated in column 1 with each variable for all continuous variables.

† Measured only at Time 1 interview.

‡ Refers to past 30 days prior to incarceration, and past 90 days after release from jail.

### Smoking patterns of participants

At the Time 1 interview, 344 of the REAL MEN participants (62.3%) were current smokers. At Time 2, 69% (N = 274) of participants reported that they were current smokers. At Time 1, 48% (N = 262) of participants were daily smokers, and 17% (N = 87) of participants reported smoking 20 cigarettes or more, e.g. a pack a day or more, prior to their incarceration. One year after the young men’s release from jail, 46% (N = 182) of participants reported being daily smokers, and 14% (N = 52) said they smoked a pack of cigarettes per day or more.

### Factors associated with smoking

At the Time 1 interview during which we assessed factors related to smoking prior to incarceration (with controls for age and report of Latino ethnicity) we found that foster care history (B = 0.91, OR = 2.49, Wald χ2 = 16.84, d.f. = 1, p = 0.000), not living with parents prior to incarceration (B = 0.77, OR = 2.16, Wald χ2 = 12.00, d.f. = 1, p = 0.001), not attending school prior to incarceration (B = 0.41, OR = 1.50, Wald χ2 = 4.51, d.f. = 1, p = 0.034), having sold drugs for drug or alcohol money (B = 0.49, OR = 1.64, Wald χ2 = 5.59, d.f. = 1, p = 0.014), number of sex partners in the three months prior to incarceration (B = 0.05, OR = 1.06, Wald χ2 = 5.51, d.f. = 1, p = 0.019), gang involvement (B = 0.46, OR = 1.59, Wald χ2 = 4.43, d.f. = 1, p = 0.035), incarceration for drug charges (B = 0.68, OR = 1.97, Wald χ2 = 10.76, d.f. = 1, p = 0.001) incarceration for violent charges (B = −0.88, OR = 0.42, Wald χ2 = 23.18, d.f. = 1, p = 0.000), and number of prior arrests (B = 0.04, OR = 1.04, Wald χ2 = 4.31, d.f. = 1, p = 0.038) were associated with the likelihood of being a smoker.

Factors associated with frequency of smoking (number of days smoked in the past 30 days) and quantity of cigarettes smoked (number of cigarettes smoked per day in the past 30 days) are shown in Table 3.

**Table 3:** factors associated with frequency and quantity of smoking prior to incarceration, N = 552

**Unstandardized regression coefficients (*betas *** **in parentheses)**		
	**Days smoked in past 30 days**	**Number of cigarettes smoked each day**
Foster care history	6.28***	2.45**
	(0.19)	(0.11)
Not living with parents or legal guardian	4.54***	2.48**
	(0.14)	(0.11)
Not attending school regularly	3.19*	1.52
	(0.10)	(0.07)
Sold drugs to get money for drugs/alcohol	3.17*	2.61**
	(0.11)	(0.14)
Sex partners in 3 mo. prior to incarceration	0.26**	0.05
	(0.12)	(0.03)
Gang involvement	2.85*	0.84
	(0.09)	(0.04)
Incarcerated for drug charges	4.55***	1.74
	(0.14)	(0.08)
Incarcerated for violent charges	−4.22***	−2.89***
	(−0.14)	(−0.14)
Prior arrests	0.35***	0.23**
	(0.14)	(0.13)

*p ≤ 0.05, **p ≤ 0.01, ***p ≤ 0.001, for t-test statistic in linear regression.

All models were adjusted for participants’ age and Latino race/ethnicity (demographic factors associated with smoking in Table 1 at p ≤ 0.05 level).

Non-smokers were coded as 0 for both measures of frequency and quantity of smoking.

One year after release from jail, report of a foster care history (B = 0.84, OR = 2.31, Wald χ2 = 8.25, d.f. = 1, p = 0.004) and number of arrests (B = 0.05, OR = 1.05, Wald χ2 = 3.78, d.f. = 1, p = 0.052) prior to incarceration were associated with current smoking, in a model that controlled for age, report of Latino ethnicity, number of days incarcerated between interviews, and randomization to the intervention. Recidivism (B = 0.37, OR = 1.45, Wald χ2 = 2.30, d.f. = 1, p = 0.129), the experience of going back to jail, was not associated with current smoking after release from jail, when controlling for age, report of Latino ethnicity, number of days incarcerated between interviews, and randomization to the intervention.

Factors associated with frequency of smoking (number of days smoked in the past 90 days) and quantity of cigarettes smoked (number of cigarettes smoked per day in the past 90 days) one year after release from jail are shown in Table 4.

**Table 4:** Factors associated with frequency and quantity of one year after release from jail, N = 397

**Unstandardized regression coefficients (*betas *** **in parentheses)**		
	**Days smoked in past 90 days**	**Number of cigarettes smoked each day**
Foster care history prior to incarceration	13.43*	3.68*
	(0.14)	(0.14)
Arrests prior to index incarceration	0.67	0.31*
	(0.09)	(0.15)
Went back to jail after index incarceration	3.11	−0.11
	(0.04)	(−0.01)

*p ≤ 0.01, for t-test statistic in linear regression.

All models were adjusted for participants’ age, Latino race/ethnicity, number of days participants were incarcerated between interviews, and whether or not they received the REAL MEN intervention.

Non-smokers were coded as 0 for both measures of frequency and quantity of smoking.

## Discussion

The smoking rates for this sample of adolescents housed in adult correctional facilities were 62.9% prior to incarceration and 69% one year after release from jail. These rates are over 4.5 times the smoking rate of Black male adolescents in the U.S., 2.5 times the smoking rate of Latino male adolescents in the U.S., and twice the smoking rate of Black adolescents in the juvenile corrections system [3,4]. There is little existing data about smoking among Latino youth in U.S. justice system. Among a comparable group New York City high-school aged youth, only 3% of Black youth and 9% of Latino youth reported smoking [23], suggesting that subpopulations of youth involved in the adult criminal justice system may be at much higher risk for smoking.

In reflecting on problem behavior theory [5,24] and our study, we did find that a cluster of early problem behaviors – drug use, sex risk, and violence– explain smoking behaviors prior to incarceration. But the absence of protective factors in our sample’s lives, like housing instability, tenuous ties to family, and criminal justice involvement, continues to influence smoking behavior over time.

Our findings reflect those of other researchers working with smoking adolescents both within [4] and outside of the justice system [6-8]. Living situation, educational circumstances, and substance use influence young people’s smoking behaviors in juvenile justice settings [4], as well as in our sample of adolescents in adult jails. Our study also demonstrated the persistent impact of foster care history on current smoking status, frequency, and quantity of smoking both prior to incarceration and after of release from jail. This finding has been documented in numerous studies of non-incarcerated samples [25-27]. Foster care history may either precipitate or compound the effect of adjudication history on smoking behaviors among young people.

Our findings about violence are somewhat contradictory, but may relate to the variables’ underlying meaning. For example, we found that being in a gang was associated with smoking. Thus, it is unclear to what extent our youth smoke in order to negotiate violent environments [28] and what role peer behavior [4,6] has in smoking during youth street organization activities. No in-depth observational studies have fleshed out this relationship. On the other hand, having violent charges was inversely associated with smoking in our study. Such charges included armed robbery, weapons possession, domestic violence, sex offenses, and simple assault. Engagement in these serious violent crimes may not represent the same kind of routine activities associated with participation in street organizations and how people navigate the “street” [29,30]. Further, in-depth study would have to occur to understand how engagement in serious violent crimes relates to smoking, and in our case not smoking. Perhaps young people engaged in violent crimes represent a unique subsample of youth who are not engaged in so-called “normative” transgressions [9], but much more serious negative behaviors that do not align with the constellation of behaviors in problem behavior theory [24].

Our finding about the role of previous arrests in smoking behaviors does substantiate the association between smoking and criminal justice history [1,2,4]. In young people’s lives, and the extent to which number of arrests is a marker of ongoing criminal justice involvement, this measure of delinquency likely speaks to the additive effect of this particular problem behavior over time. In other words, problem behaviors that occur often may have unique implications for other problem behaviors. In practical terms, venues through which delinquent youth move may be sensible places to recruit for interventions.

### Intervention and public health policy implications

Problem behavior theory offers that adolescent risk behaviors, like smoking, are not only normative, but also may serve a function, have purpose, and be goal-directed [24]. Therein lies the opportunity for intervention. Jessor argues that, for example, a behavior like smoking can help adolescents gain respect, acceptance, establish autonomy, be linked to repudiation of norms, may mark a transition from childhood to adulthood, and may be instrumental in helping young people cope with anxiety, frustration, or disappointment [24]. Risk behaviors occur because there may be no viable alternatives to help young people reach these goals and satisfy these important functions.

Future research must address the functionality of smoking in young men’s lives. Is it used to negotiate “the street,” for example drug deals? Is it used to negotiate discrimination and feelings of alienation? Or is it used to manage the stress, anxiety, and depression that stems from the social context in which our sample of arguably disadvantaged young me live in? Research that probes into the functions of smoking could offer clues as to how to intervene, for example, with harm reduction approaches that give young people other types of skills and activities that facilitate their ability to navigate their neighborhood, deal with feelings of discrimination, or comprehensive and accessible mental health treatment options to address psychological distress stemming from housing or familial instability.

Those who have tested problem behavior theory empirically have also found that smoking has more to do with a clustering of problem behaviors, than it has to do with concern for health among adolescents [9]. Thus, interventions have to address the cluster of problem behaviors simultaneously, rather than appeal to the youth’s sense (or lack thereof) of health promotion. For example, interventions would simultaneously address drug, sex, and risk for delinquency using a harm reduction approach, rather than isolating one health problem using a health promotion strategy [11,17]. These interventions could also be tailored along gender, race, and class lines, as well as be specific to how people navigate their social context [11,24]. In our sample’s case, findings may also give specific clues as to where we might intervene, e.g. early on in foster care or using comprehensive risk reduction messaging in detention facilities with youth.

From a public health policy perspective, researchers and advocates might begin to target the institutions through which many disadvantaged youth cycle, for example foster care agencies, juvenile detention centers, jails, in mental health or drug treatment programs, alternative schools, GED, or employment programs. Many adolescent smoking prevention programs have occurred at the school level [19,21], reaching those students who are most likely to succeed. By targeting the institutions that reach those most at risk with advocacy efforts, funding initiatives, and interventions, public health practitioners may have an impact on the highest risk youth. Such interventions are certainly possible [20].

### Study limitations

This study had several limitations. First, because this was a secondary analysis of a study whose goal was not to reduce tobacco use, our measures of smoking behavior, as well as predictor variables, may not have been as appropriate as a study that would be designed with the goal of examining tobacco use. For example, rather than self-report, biochemical verification of smoking may have been appropriate. We might have included specific measures to test a theory of problem behaviors. A second limitation is attrition in this study. Though there were few differences at baseline between those who completed the follow-up interview and those who did not, we have no way of knowing what the circumstances were for the 28% (N = 155) of participants for whom we lost. A third limitation of our study is the specific nature of our sample – mostly Black and Latino adolescents who were incarcerated in adult jails. The incarceration of young people in adult jails is a unique policy and may have implications for generalizability of our findings, as well as the racial and ethnic makeup of the jurisdiction that we were studying. Nevertheless, ours is one of a handful of studies to focus on the smoking behaviors of adjudicated young people, offering insights into this high-risk group of young men’s experiences prior to incarceration and after release from jail.

## Conclusion

A cluster of problem behaviors, including drug risk, sex risk, violence, and criminal justice involvement, may help explain adolescents’ smoking behaviors prior to incarceration. Upon release from jail and as adolescents transition into adulthood, however, their histories of difficult life circumstances and ongoing criminal justice involvement may have long-term consequences for smoking behaviors. Findings from our study suggest the need for in-depth research about the functionality of smoking in the lives of disadvantaged young people. Ultimately, comprehensive risk reduction strategies are needed that address a range of problem behaviors, the social context in which high-risk young men live, as well as the settings in which these young men become institutionalized.

## Competing interests

The authors declare that they have no competing interests.

## Authors’ contributions

MR oversaw all stages of the manuscript and wrote the final draft of the manuscript as well as revisions of the manuscript. She also conducted all analyses. BF wrote and revised the introduction. KC revised drafts of the manuscript. MJ and KD wrote the first draft of the introduction and methods sections of the manuscript. NF consulted on the design of the manuscripts and revised several drafts. He also served as Principal Investigator of the study in the manuscript. All authors read and approved the final manuscript.
